# The association between caregiver distress and individual neuropsychiatric symptoms of dementia

**DOI:** 10.1590/S1980-57642013DN70300009

**Published:** 2013

**Authors:** Annibal Truzzi, Letice Valente, Eliasz Engelhardt, Jerson Laks

**Affiliations:** 1 MD, PhD. Centro de Estudos e Pesquisa do Envelhecimento, Instituto Vital Brasil e Centro para Pessoas com Doença de Alzheimer, Instituto de Psiquiatria da Universidade Federal do Rio de Janeiro.; 2 Centro para Pessoas com Doença de Alzheimer, Instituto de Psiquiatria da Universidade Federal do Rio de Janeiro.; 3 MD, PhD. Cognitive and Behavioral Neurology Unit – INDC-CDA/IPUB – Universidade Federal do Rio de Janeiro.; 4 MD, PhD. Centro para Pessoas com Doença de Alzheimer, Instituto de Psiquiatria da Universidade Federal do Rio de Janeiro.

**Keywords:** neuropsychiatric symptoms, caregiver distress, Alzheimer's disease, dementia

## Abstract

Neuropsychiatric symptoms (NPS) of dementia constitute one of the most related factors to caregiver burden and patients' early institutionalization. Few studies in Brazil have examined which symptoms are associated with higher levels of caregiver distress.

**OBJECTIVE:**

To evaluate the caregiver distress related to individual NPS in familial caregivers of patients with dementia. We also examined which caregiver and patient factors predict caregiver distress associated with NPS.

**METHODS:**

One hundred and fifty-nine familial caregiver and dementia outpatient dyads were included. The majority of the patients had a diagnosis of Alzheimer's disease (66.7%). Caregivers were assessed with a sociodemographic questionnaire, Beck Anxiety and Depression Inventories, and the Neuropsychiatric Inventory – Distress Scale. Patients were submitted to the Mini-Mental State Examination, Functional Activities Questionnaire, and the Neuropsychiatric Inventory. Spearman's rank correlation was used to assess the relationships between the continuous variables. Multiple linear regression analyses with backward stepping were performed to assess the ability of caregiver and patient characteristics to predict levels of caregiver distress associated with NPS.

**RESULTS:**

Apathy (M=1.9; SD=1.8), agitation (M=1.3; SD=1.8), and aberrant motor behavior (AMB) (M=1.2; SD=1.7) were the most distressful NPS. The frequency/severity of NPS was the strongest factor associated with caregiver distress (rho=0.72; p<0.05).

**CONCLUSION:**

The early recognition and management of apathy, agitation and AMB in dementia patients by family members and health professionals may lead to better care and quality of life for both patients and caregivers.

## Abstract (pt)

Os sintomas neuropsiquiátricos (SNP) da demência constituem um dos fatores mais relacionados à sobrecarga do cuidador e à institucionalização precoce do paciente. Poucos estudos brasileiros examinaram quais sintomas estão associados ao desgaste do cuidador.

**OBJETIVO:**

Avaliar o desgaste referente aos SNP individuais cuidadores familiares dos pacientes com demência. Nós também analisamos quais fatores dos pacientes e dos cuidadores constituem fortes preditores ao desgaste do cuidador.

**MÉTODOS:**

Cento e cinquenta cuidadores familiares e pacientes foram incluídos. A maioria dos pacientes possuía um diagnóstico de doença de Alzheimer (66,7%). Os cuidadores foram avaliados através de um questionário sociodemográfico, os Inventários de Ansiedade e Depressão de Beck e o Inventário Neuropsiquiátrico – Desgaste. Os pacientes foram submetidos ao Mini-Exame do Estado Mental, Escala de Atividades Funcionais e ao Inventário Neuropsiquiátrico. A correlação de Spearman foi utilizada para avaliar as relações entre as variáveis contínuas. Análises regressivas lineares múltiplas foram realizadas para analisar, dentre as variáveis dos pacientes e dos cuidadores, os fatores preditores do desgaste do cuidador.

**RESULTADOS:**

Apatia (M=1,9; DP=1,8), agitação (M=1,3; DP=1,8) e comportamento aberrante motor (CMA) (M=1,2; DP=1,7) foram os SNP mais desgastantes. A frequência/gravidade dos SNP foi o fator mais fortemente associado ao desgaste do cuidador (rho=0.72; p<0,05).

**CONCLUSÃO:**

O reconhecimento precoce e manejo da apatia, agitação e CMA nos pacientes com demência pelos familiares e profissionais de saúde possivelmente levará a uma melhor qualidade vida e cuidado para pacientes e cuidadores.

## INTRODUCTION

Neuropsychiatric symptoms (NPS) of dementia constitute one of the most related factors to caregiver burnout and early institutionalization of patients.^1,2^ Different subtypes of NPS impose varied patterns of distress experienced by the caregiver. Aberrant Motor Behavior (AMB) and sleep disturbances are highly distressing because they impose higher physical demands. Delusions of theft and identification are psychologically stressful for the caregiver because of the patient's inability to recognize them.

The burden associated with NPS is also influenced by the stage of the dementia. Depressive and anxious symptoms are commonly observed during the early stages of Alzheimer's disease (AD), whereas psychotic symptoms and AMB occur in the moderate stages of the dementia.^3^

Lack of familial and social support also contributes to the stress associated with NPS. In developing countries, lack of knowledge about NPS by caregivers and lack of health facilities that may assist patients with these symptoms increase the distress of familial caregivers.^4^

The financial burden associated with NPS constitutes an important source of caregiver stress. The study conducted by Murman et al.^5^ which included 128 caregiver and AD patient dyads, estimated that a one-point increase in the Neuropsychiatric Inventory (NPI) score would result in an annual increase of between US$247 and US$409 in total direct costs.

Inconsistent findings in the literature fail to point out which NPS are more distressful to caregivers. However, agitation, anxiety, delusions and sleep disturbances are among the most reported stressful symptoms.^6-8^ There is a paucity of studies conducted in Brazil evaluating the distress associated with NPS of familial caregivers of patients with dementia.

The aim of this study was to evaluate the distress related to individual NPS among familial caregivers of patients with dementia. Also, an examination into which caregiver and patient factors are the strongest predictors of caregiver distress associated with NPS was carried out.

## METHODS

**Sample.** This is a cross-sectional study. The evaluations were carried out by a multiprofessional team that consisted of geriatric psychiatrists and neuropsychologists. A comprehensive dementia evaluation was performed in all patients which included a history with information gathered from patients and caregivers, a brief neuropsychological test battery, physical examination, blood tests, and a Computed Tomography or Magnetic Resonance Imaging scan of the brain.

The study sample comprised 159 caregiver and dementia outpatient dyads treated at the Centre for Alzheimer's Disease of the Federal University of Rio de Janeiro. Patients were predominantly female (N=107; 67.3%), had finished college (N=51; 31.9%), and had a mean age of 76.9 (SD=6.9) years. The study sample included 106 (66.7%) patients with a diagnosis of either possible or probable AD according to the National Institute of Neurological and Communicative Disorders and Stroke – Alzheimer's disease and Related Disorders Association,^9^ twenty three (14.5%) with Vascular Dementia (VaD) according to the National Institute of Neurological Disorders and Stroke and Association Internationale pour la Recherche et l'Enseignement en Neuroscience,^10^ and thirty (18.8%) individuals with Mixed Dementia according to the Diagnostic and Statistical Manual of Mental Disorders – Fourth Edition.^11^

The familial caregivers who provided the information regarding patients' NPS had to be older than 18 years and have at least weekly face-to-face contact with the patient. The majority of the caregivers were middle-aged daughters of the patients, with a mean time as caregiver of 3.8 (SD=2.7) years.

**Measurements. Patients.** *Neuropsychiatric Inventory (NPI) –* The NPI assesses 10 NPS, namely: delusions, hallucinations, depression/dysphoria, anxiety, agitation/ aggression, euphoria, disinhibition, irritability/lability, apathy and AMB. Severity and frequency are independently assessed and the total score is given as frequency x severity with a maximum score of 120 indicating the worst score. The Brazilian validated version of the NPI was applied.^8^

*Mini-Mental State Examination (MMSE) –* The MMSE evaluates global mental function and consists of 30 items assessing five cognitive domains, with a maximum score of 30 points: orientation to time and place (10 points), registration of 3 words (3 points), attention and calculation (5 points), recall of 3 words (3 points) and language (8 points). The MMSE version used in this study was validated for Brazilian Portuguese by Bertolucci et al.^12^

*Functional Activities Questionnaire (FAQ) –* The FAQ consists of a 10-item scale that assesses instrumental activities of daily living (IADL) and basic functional capacities in older people, with each rated on a four-point scale (0 being normal and 3 incapable) giving a maximum score of 30 points (severely disabled).^13^ The FAQ has been systematically used in other studies conducted in Brazil.^14,15^

**Caregivers.** *Sociodemographic Questionnaire –* A brief self-administered sociodemographic questionnaire was developed by the authors in order to collect general sociodemographic data such as age, gender, marital status, family relationship, level of schooling and time as caregiver.

*Neuropsychiatric Inventory - Distress Scale (NPI-D) –* The NPI-D consists of an instrument evaluating caregiver distress related to 10 individual NPS measured by the NPI. Distress intensity is scored from 0 (none) to 5 (very intense or extreme). The total score of the NPI-D is obtained by summing the scores on each of its subitems.^8,16^

*Beck Depression Inventory (BDI) –* The BDI is a 21-item self-rating scale that covers a variety of depressive symptoms including feelings of sadness, concerns about the future, suicidal ideation, tearfulness, sleep, fatigue, interests, worries about health, sexual interest, appetite, weight loss and general enjoyment. Each item is rated as 0, 1, 2 and 3 denoting increasing severity of symptoms. The Brazilian version of the BDI validated by Gorenstein and Andrade^17^ was employed.

*Beck Anxiety Inventory (BAI) –* The BAI consists of a self-administered instrument with 21 items covering the most frequent anxiety symptoms seen in clinical practice. Each item is scored 0, 1, 2 or 3, with higher scores denoting increasing severity of symptoms. The validated Brazilian Portuguese version of the BAI was administered.^18^

**Statistical analysis.** All variables were inspected for normality before further analyses. Results were expressed as mean (M) and standard deviations (SD). Spearman's rank correlation was used to assess the relationships between the continuous variables, such as the NPI-D and each sub-item score, and caregiver and patient variables.

Multiple linear regression analyses with backward stepping were performed to assess the ability of caregiver and patient characteristics to predict levels of caregiver distress associated with NPS.

Data were analyzed using the SPSS statistical package version 19.0.

**Ethical issues.** A full description of the study was given to patients and their caregivers. Explicit consent was required for enrolment, and the caregivers were asked to sign the informed consent form stating they agreed to disclose the patient's clinical and sociodemographic information. This procedure was approved by the Research Ethics Committees of the Federal University of Rio de Janeiro.

## RESULTS

The characteristics of the caregivers and patients are shown in Table 1. Most of the patients had a diagnosis of AD (66.7%) of moderate severity according to the cognitive and the functional evaluations.

**Table 1:** Characteristics of the 159 caregiver and outpatient dyads.

**Caregiver characteristics**		
Females (%)		131 (82)
Adult children (%)		88 (56)
Spouses (%)		45 (28)
Other family members (%)		26 (16)
Age, mean (SD)		56.2 (13.3)
Time as caregiver, mean (SD)		3.8(2.7)
Finished college (%)		51 (31.9)
Married (%)		87 (54.4)
BAI, mean (SD)		8.8 (8.9)
BDI, mean (SD)		10.5 (8.9)
NPI-D, mean (SD)		9.4 (8.5)
**Patient characteristics**		
Female (%)		107 (67)
Age, mean (SD)		76.9 (6.9)
MMSE, mean (SD)		14.9 (6.8)
FAQ, mean (SD)		20.6 (8.2)
NPI, mean (SD)		21.4 (18.6)
Diagnosis (%)	Alzheimer's disease	106 (66.7)
	Vascular dementia	23 (14.5)
	Mixed dementia	30 (18.8)

SD: standard deviation; BAI: Beck Anxiety Inventory; BDI: Beck Depression Inventory; MMSE: Mini-Mental State Examination; FAQ: Functional Activities Questionnaire; NPI-D: Neuropsychiatric Inventory – Distress Scale; NPI: Neuropsychiatric Inventory.

**Figure 1:**
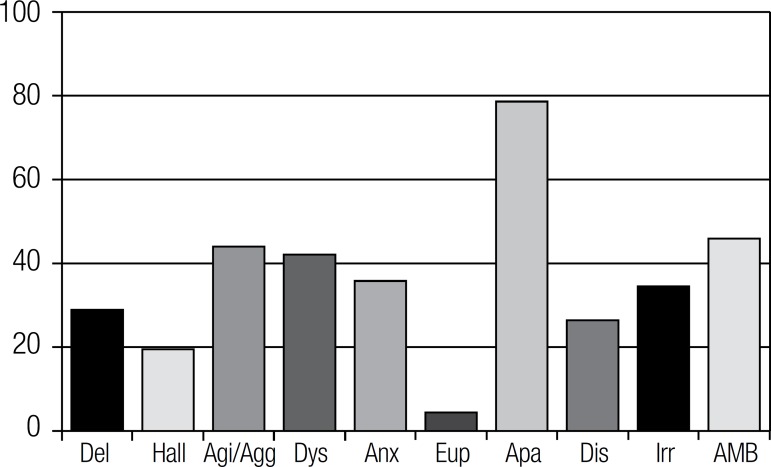
Prevalence of neuropsychiatric symptoms (%) (N=159).

The frequencies of NPS as measured by the NPI are reported in Figure 1. Apathy (78.6%), AMB (45.9%) and agitation (44%) were the most common symptoms in the sample.

**Figure 2:**
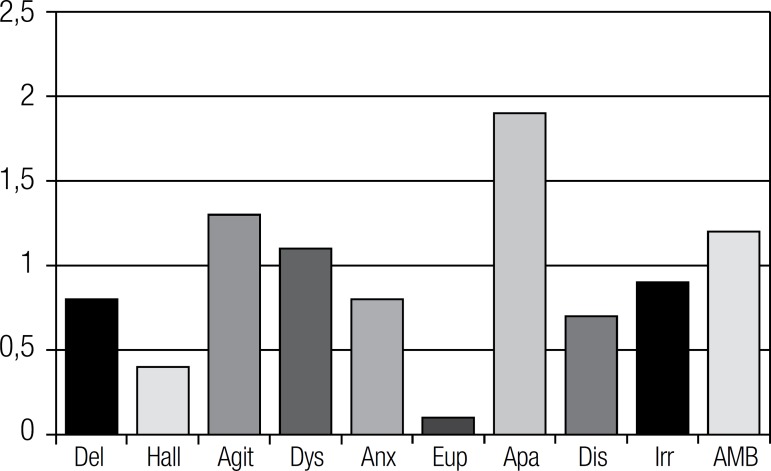
Caregiver distress according to individual NPS (mean) (N=159).

The most distressful NPS for caregivers were apathy (M=1.9; SD=1.8), agitation (M=1.3; SD=1.8), and AMB (M=1.2; SD=1.7), according to the results in Figure 2.

A strong correlation was found between NPI and NPI-D total scores (Spearman rho=0.72; p<0.05). The correlation analyses also revealed strong correlations between individual NPS according to NPI subscores and caregiver distress as measured by NPI-D subscores (Spearman rho >0.70; p<0.05).

It was decided to exclude the NPI total score from the linear regression analyses due to the high correlation with NPI-D. Using NPI-D as the dependent variable, and taking caregiver and patient characteristics as explanatory variables, four variables remained in the linear regression analyses. These variables were responsible for an explained variance of 8.5%. Scores on the MMSE (β=0.25, p<0.05), FAQ (β=0.30, p<0.01), and BDI (β=0.17, p<0.05), as well as time as caregiver (β= –0.15, p<0.05), were significantly associated with NPI-D.

## DISCUSSION

This study showed that apathy, agitation, and AMB are the most distressful NPS according to familial caregivers of patients with dementia. We also found that among caregiver and patient characteristics, the frequency and severity of NPS was the strongest factor associated with caregiver distress.

The majority of the caregivers in our sample were the daughters of the patients, followed by the spouses. This finding is consistent with previous studies conducted in Brazil, which found that daughters are the primary caregivers of patients with dementia.^19,20^

Apathy was the most prevalent neuropsychiatric symptom in our sample, comprising predominantly moderate AD outpatients. Other studies investigating the prevalence of NPS in AD outpatients also found apathy to be the most frequent symptom.^8,21,22^

Our study found a frequency of agitation similar to the rate found in a previous study conducted in Brazil (44%).^23^ Higher prevalences of agitation were found in two Brazilian studies, conducted by Vega et al.^22^ (58%) and Camozzato et al.^8^ (62%).

The frequency of AMB in our sample is comparable to that found by Cammozzato et al.^8^ (45%). Other studies conducted in Brazil have shown mixed results for AMB prevalence in outpatients with dementia. Thirty percent of the sample from the study conducted by Balieiro Jr. et al.^23^ showed AMB, whereas 68% of patients from the study performed by Vega et al.^22^ presented with this symptom.

Apathy can be defined as an internal state of lack of interest or behavioral inaction. It comprises a spectrum of symptoms that includes reduced initiative, interest, motivation, energy, and enthusiasm.^24^ Apathetic behavior is a major source of distress for those who take care of patients with dementia at home.^25^ Likewise in our study, apathy was the most distressful neuropsychiatric symptom for the familial caregivers. A similar finding was obtained in the studies conducted by Camozzato et al.^8^ and Kaufer et al.^16^. The association between apathy and caregiver distress may be explained by the higher disability it imposes on patients and the promotion of a sense of frustration in caregivers.

Previous studies conducted in Latin America show that AMB is one of the most distressful neuropsychiatric symptom for familial caregivers.^8,26^ In our study, it was the second-most distressful symptom. Repetitive behaviors, including wandering and vocalization, tend to be misinterpreted as deliberate misbehavior by familial caregivers from Latin America.^4^ This lack of knowledge about AMB by caregivers may contribute to increased levels of distress in this group.

More than half of AD patients exhibit agitated behavior in the course of their illness.^27^ This includes an array of problematic behaviors such as aggression, resistive behaviors, threats, shouting, and cursing. Agitation is significantly associated with psychosis, depression and diurnal behavioral disturbances in AD patients.^28,29^ Patients with agitation demand more physical effort from the caregivers. This may explain why agitation has figured as one of the most common NPS related with caregiver distress in studies that have included patients with dementia.^8,16,23^

The strong correlation found between NPI total score and NPI-D lends further support for the close relationship between NPS and caregiver distress. Kaufer et al.^16^ also found a strong correlation (r=0.64; p<0.01) between NPS and caregiver distress. A systematic review conducted by Black and Almeida^30^ showed that more than half of the 11 studies (N=8) examining correlations between NPS and caregiver stress found strong correlations between these two variables (r>0.40; p<0.01).

The present study has limitations which should be acknowledged. First, our study has a cross-sectional design that does not allow us to make causal inferences. Longitudinal studies are necessary to clarify how NPS lead to caregiver distress. The overrepresentation of AD patients in our sample is probably due to the fact that patients with dementia of other etiologies are referred to different specialists, such as neurologists. However, we believe that our sample is representative overall of patients with dementia that regularly attend psychogeriatric facilities in Brazil. NPS describe an array of symptoms which tend to fluctuate with time and that may be challenging to separate in clinical practice. Furthermore, neuropsychiatric subsyndromes with symptoms that tend to occur together have been proposed, with distinct neurobiological correlates.^28^ Further studies involving larger samples are necessary to elucidate which subsyndromes are more distressful for caregivers. Finally, the fact that our sample is drawn from a psychogeriatric facility precludes generalization of our findings.

To sum up, apathy, AMB, and agitation represent a major source of distress to familial caregivers who take care of patients with dementia. Early recognition of these particular symptoms by family members and health professionals, and prompt implementation of different treatment strategies, may lead to better care and quality of life for both patients and caregivers.

## References

[1] Truzzi A, Valente L, Ulstein I, Engelhardt E, Laks J, Engedal K (2012). Burnout in familial caregivers of patients with
dementia. Rev Bras Psiquiatr.

[2] Gauthier S, Cummings J, Ballard C (2010). Management of behavioral problems in Alzheimer’s
disease. Int Psychogeriatr.

[3] Desai AK, Schwartz L, Grossberg GT (2012). Behavioral disturbance in dementia. Curr Psychiatry Rep.

[4] Ferri CP, Ames D, Burns A (2004). Behavioral and psychological symptoms of dementia in developing
countries. Int Psychogeriatr.

[5] Murman DL, Chen Q, Powell MC, Kuo SB, Bradley CJ, Colenda CC (2002). The incremental direct costs associated with behavioral symptoms
in AD. Neurology.

[6] Fuh JL, Liu CK, Mega MS, Wang SJ, Cummings JL (2001). Behavioral disorders and caregivers’ reaction in Taiwanese
patients with Alzheimer’s disease. Int Psychogeriatr.

[7] Matsumoto N, Ikeda M, Fukuhara R (2007). Caregiver burden associated with behavioral and psychological
symptoms of dementia in elderly people in the local
community. Dement Geriatr Cogn Disord.

[8] Camozzato AL, Kochhann R, Simeoni C (2008). Reliability of the Brazilian Portuguese version of the
Neuropsychiatric Inventory (NPI) for patients with Alzheimer’s disease and
their caregivers. Int Psychogeriatr.

[9] McKhann G, Drachman D, Folstein M, Katzman R, Price D, Stadlan EM (1984). Clinical diagnosis of Alzheimer’s disease: report of the
NINCDSADRDA Work Group under the auspices of department of health and human
services task force on Alzheimer’s disease. Neurology.

[10] Román GC, Tatemichi TK, Erkinjuntti T (1993). Vascular dementia: diagnostic criteria for research studies.
Report of the NINDS-AIREN International Workshop. Neurology.

[11] American Psychiatric Association (1994). Diagnostic and Statistical Manual of Mental Disorders.

[12] Bertolucci PHF, Brucki SMD, Campacci SR, Juliano Y (1994). O mini exame do estado geral em uma população geral
– impacto da escolaridade. Arq Neuropsiquiatr.

[13] Pfeffer RI, Kurosaki TT, Harrah CH, Chance JM, Filis S (1982). Measurement of functional activities in older adults in the
community. J Gerontol.

[14] Marra TA, Pereira LSM, Faria CDCM, Pereira DS, Martins MAA, Tirado MGA (2007). Avaliação das atividades de vida diária de
idosos com diferentes níveis de demência. Rev Bras Fisioter.

[15] Laks J, Baptista EMR, Contino ALB, de Paula EO, Engelhardt E (2007). Mini-Mental State Examination norms in a community-dwelling
sample of elderly with low schooling in Brazil. Cad Saude Publica.

[16] aufer DI, Cummings JL, Christine D (1998). Assessing the impact of neuropsychiatric symptoms in Alzheimer’s
disease: the Neuropsychiatric Inventory Caregiver Distress
Scale. J Am Geriatr Soc.

[17] Gorestein C, Andrade L (1996). Validation of a Portuguese version of the Beck Depression
Inventory and the State-Trait Anxiety Inventory in Brazilian
subjects. Braz J Med Biol Res.

[18] unha JA (2001). Manual das versões em português das escalas Beck.

[19] Garrido R, Menezes PR (2004). Impact on caregivers of elderly patients with dementia treated at
a psychogeriatric service. Rev Saude Publica.

[20] Cassis SVA, Karnakis T, Moraes TA, Curiati JAE, Quadrante ACR, Magaldi RM (2007). Correlation between burden on caregiver and clinical
characteristics of patients with dementia. Rev Assoc Med Bras.

[21] Lyketsos CG, Steinberg M, Tschanz JT, Norton MC, Steffens DC, Breitner JC (2000). Mental and behavioral disturbances in dementia: findings from the
Cache County Study on Memory in Aging. Am J Psychiatry.

[22] Vega UM, Marinho V, Engelhardt E, Laks J (2007). Sintomas neuropsiquiátricos nas demências: relato
preliminar de uma avaliação prospectiva em um
ambulatório do Brasil. Arq Neuropsiquiatr.

[23] Balieiro Jr. AP, Sobreira EST, Pena MCS, Silva-Filho JH, Vale FAC (2010). Caregiver distress associated with behavioral and psychological
symptoms in mild Alzheimer’s disease. Dement Neuropsychol.

[24] Tagariello P, Girardi P, Amore M (2009). Depression and apathy in dementia: same syndrome or different
constructs?. A critical review. Arch Gerontol Geriatr.

[25] Brodaty H, Burns K (2012). Nonpharmacological management of apathy in dementia: a systematic
review. Am J Geriatr Psychiatry.

[26] Mangone CA, Bueno AA, Allegri R (2000). Behavioral and psychological symptoms of dementia in Latin
America. Int Psychogeriatr.

[27] Bergh S, Engedal K, Roen I, Selbaek G (2011). The course of neuropsychiatric symptoms in patients with dementia
in Norwegian nursing homes. Int Psychogeriatr.

[28] Truzzi A, Ulstein I, Valente L (2013). Patterns of neuropsychiatric sub-syndromes in Brazilian and
Norwegian patients with dementia. Int Psychogeriatr.

[29] Aarsland D, Cummings JL, Yenner G, Miller B (1996). Relationship of aggressive behavior to other neuropsychiatric
symptoms in patients with Alzheimer’s disease. Am J Psychiatry.

[30] Black W, Almeida OP (2004). A systematic review of the association between the Behavioral and
Psychological Symptoms of Dementia and burden of care. Int Psychogeriatr.

